# Road safety, health equity, and the built environment: perspectives of transport and injury prevention professionals in five Canadian municipalities

**DOI:** 10.1186/s12889-023-16115-7

**Published:** 2023-06-22

**Authors:** Emily McCullogh, Alison Macpherson, Brent Hagel, Audrey Giles, Pamela Fuselli, Ian Pike, Juan Torres, Sarah A. Richmond

**Affiliations:** 1 York University, Toronto, Canada; 2 University of Calgary, Calgary, Canada; 3 University of Ottawa, Ottawa, Canada; 4 Parachute, Ottawa, Canada; 5 University of British Columbia, Vancouver, Canada; 6 University of Montreal, Montreal, Canada; 7 Public Health Ontario, Ontario, Canada

**Keywords:** Injury prevention, Road safety, Built environment, Vulnerable road users, Health equity, Public health

## Abstract

**Background:**

Concerns regarding health equity (HE) and the built environment (BE) are well established in the Canadian urban context. Transport and injury prevention professionals across sectors, such as transportation and public health, are involved in designing and implementing BE interventions that enhance the safety of vulnerable road users (VRUs). Results from a larger study examining barriers and facilitators to BE change are used to illustrate how transport and injury prevention professionals perceive HE concerns in their work in five Canadian municipalities. Broadening our understanding of how HE influences the professional BE change context is crucial when advocating for modifications that enhance the safety of equity-deserving VRUs and groups who experience marginalization.

**Methods:**

Interview and focus group data were gathered from transport and injury prevention professionals working in policy/decision-making, transport, police services, public health, non-profit organizations, schools/school boards, community associations, and private sectors across five Canadian urban municipalities: Vancouver, Calgary, Peel Region, Toronto, and Montréal. Data were analyzed using thematic analysis (TA) to illustrate how equity considerations were perceived and applied in participants’ BE change work.

**Results:**

The results of this study illustrate transport and injury prevention professionals’ awareness of the varying needs of VRUs, as well as the inadequacies of current BEs in the Canadian urban context and consultation processes utilized to guide change. Participants emphasized the importance of equitable community consultation strategies, as well as specific BE changes that would support the health and safety of VRUs. Overall, the results highlight how HE concerns inform transport and injury prevention professionals’ BE change work in the Canadian urban context.

**Conclusion:**

For professionals working in urban Canadian transport and injury prevention sectors HE concerns influenced their perspectives of the BE and BE change. These results illustrate a growing need for HE to guide BE change work and consultation processes. Further, these results contribute to ongoing efforts in the Canadian urban context to ensure that HE is at the forefront of BE policy change and decision-making, while promoting existing strategies to ensure that the BE, and related decision-making processes, are accessible and informed by a HE lens.

## Introduction/Background information

The health and wellness of Canadians is shaped by conditions in which they live [1, 2]. This includes the design of the built environment (BE), which refers to “the human-made surroundings that provide the setting for all human activity, including those places where people live, work, learn, rest, and play” [3]. Further, transportation and mobility infrastructure “form the connective tissue that links these places together and represents an integral part of the built environment” [4]. Considered a modifiable determinant of health [3], the BE influences people’s active transportation (AT) habits and safety when walking, cycling, or navigating Canadian roadways as a vulnerable road user (VRU) without a motor vehicle [5, 6]. Further, there are increased injury risks for specific VRU groups, such as children, older adults, pedestrians, and cyclists [7]. Given how road users’ health and safety is dependent on the BE [8], system designers, as well as policy and decision-makers, have an obligation to make changes that reduce road users’ vulnerability and risk of injury.

The role of the BE in health and injury prevention are well established [9], particularly with regards to rates of injury [10–12] and active travel [13, 14]. Research illustrates that “modifying the built environment can help to prevent many types of injuries” [12], including motor vehicle collisions (MVCs) [10]. In addition, changes to the BE can “influence the speed of travelers and the complexity of the conflicts they are exposed to in the environment” [8], which reduces crash risk. Traffic calming interventions are an example of successful BE change that reduce road traffic injury rates, particularly in high-income countries and neighbourhoods [15]. Further, the design of the BE can increase rates of AT [5, 6], as well as access to green spaces and healthier food options [3]. Overall, the design of the BE is a critical factor influencing people’s health through transportation and urban development [16] and the disproportionate rates of injury and death experienced by VRUs requires that this public health issue be examined through the lens of health equity.

A notable gap in this body of literature is the lack of research examining the links between road safety, health equity (HE), and BE change work from the perspectives of professionals working in transport and injury prevention sectors in Canada. Professionals in these roles are at the frontlines of BE change decision-making and implementation within their municipalities and their position offers unique insight into the barriers and facilitators to equitable BE changes [17], which can be used to guide future work and policy changes. Given the established links between the BE and health, the plethora of research highlighting connections between the BE, socioeconomic status (SES), and health [3, 14], as well as the lack of BE change in equity-deserving [18] neighborhoods [19, 20], increasing our understanding of the barriers to implementing BE change through the lens of health equity is necessary.

### Health equity (HE)

According to the World Health Organization (WHO) equity is defined as “the absence of unfair, avoidable or remedial differences among groups of people, whether those groups are defined socially, economically, demographically, or geographically by other dimensions of inequality (e.g., sex, gender, ethnicity, disability, or sexual orientation)” [21], and asserts health as a fundamental human right. Implementing HE involves the elimination of inequalities and “removing obstacles to health – such as poverty and discrimination and their consequences, which include powerlessness and lack of access to good jobs with fair pay; quality education, housing, and health care; and safe environments” [22]. In other words, health inequalities “are the products of disparities in exposure to health-harming and health-promoting conditions, which themselves are the products of disparities in access to key resources and opportunities” [23].

A HE approach to BE change highlights these concerns and emphasizes the obligations of policy and decision-makers to create safe environments that support the health and reduces injury rates of equity-deserving [18] groups. As a primary concern across public health sectors, “it has become a key term in discourses about improving health and wellbeing of marginalized and racialized peoples” [24]. An example in the Canadian context is the Ontario Public Health Standards, Health Equity Guideline [25], which outlines specific requirements to help boards of health engage in public health practices that increase opportunities for optimal health amongst socially disadvantaged and equity-deserving [18] groups. Multi-sectoral collaboration is considered crucial for implementing public health interventions and advancing healthy public policies, which has been identified as a significant facilitator for BE change [17]. Further, structural determinants such as political leadership, financial commitment, and public engagement [26] influence how capacity and resources are distributed. Intervening on these determinants to create healthier environments for all road users is a public health issue and requires equity-informed transformations to the BE.

### HE and the BE

The structural design of the BE directly impacts the health of its users [27]. As noted above, Braveman and colleagues emphasize the importance of “safe environments” as a key factor in people’s health, which further emphasizes the link between the BE and road users’ safety [22]. However, the design of BEs prioritize the needs of some users over others [27, 28]. For example, motor vehicle users are prioritized in the design of the BE in Canadian cities [17], which significantly contribute to health inequities. Further, BE modifications that reduce the risk of pedestrian MVCs are more commonly implemented in high SES neighbourhoods [19] and not implemented equally across communities [27, 28], which draws attention to the power of decision-makers and their obligation to ensure the health and safety of all road users. In other words, through the lens of HE, decisions about the locations of BE modifications reflect significant inequities in our transportation systems, specifically, and society, broadly.

The exacerbated risks disproportionately experienced by VRUs on Canadian roadways amplifies the need for change. In this paper we utilize data from a pan-Canadian study examining barriers and facilitators to BE change across five urban regions in Canada (Vancouver, Calgary, Peel Region, Toronto, and Montréal) [17] and highlight data pertaining to equity and accessibility. Gaining more fulsome knowledge about equitable BE changes, the perspectives of professionals working to enact these changes, and the consequences of their absence is necessary to support future policy changes in the Canadian road safety context.

## Methods

Qualitative data were collected for a pan-Canadian study examining collision and AT rates in five Canadian cities: Vancouver, Calgary, Peel Region, Toronto, and Montréal. The question guiding this research was, *what are the barriers and facilitators to BE change?* While the scope of the broader research project did not specifically ask about HE, our analysis illuminated significant themes about the accessibility and equity of the BE. These results are significant and emphasized the need for changes not only to the geometric features of the BE, but also to BE change decision-making and implementation to ensure that VRUs and groups who experience marginalization are represented.

### Participant recruitment and data collection

Participants included professionals working in the fields of transport and injury prevention and were recruited using purposeful [29] and snowball sampling [30] techniques. Participants were recruited through research team members’ professional contacts via e-mail, Twitter, or LinkedIn across different sectors: policy/decision-making, transportation, police services, public health, university researchers, non-profit organizations, schools/school boards, community associations, and private sectors (Table 1).

**Table 1:** Number of participants by municipality and sector

	Vancouver	Calgary	Peel	Toronto	Montréal	Total
**Policy/Decision-maker** (current and former city councillors; chiefs of staff; policy advisors)	0	3	1	1	5	**10**
**Transportation** (managers of traffic operations; traffic safety analysts; traffic and transportation engineers; transportation project managers)	2	2	6	4	1	**15**
**Police Services** (traffic operations)	0	0	0	2	0	**2**
**Public Health** (chronic disease and injury prevention specialists; health promoters and planners; BE specialists)	2	2	5	4	1	**14**
**University Researcher** (population health)	1	0	0	0	3	**4**
**Non-profit** (active travel; sustainable neighbourhoods; school travel planning)	4	6	0	3	7	**20**
**Schools/ Schoolboard** (teachers; administrative staff)	3	1	0	3	3	**10**
**Community Association** (active and safe travel programs)	0	1	0	0	0	**1**
**Private** (road safety; engineer)	1	1	0	1	1	**4**
**Total**	**13**	**16**	**12**	**18**	**21**	**80**

Semi-structured key informant (KI) interviews (n = 42) were conducted from January to December 2019 by research team members in each city (Table 2). Interviews followed a semi-structured guide and were conducted one-on-one, with the exception of Calgary (n = 1) and Peel Region (n = 3) where interviews were conducted with two participants. Interviewers attended group meetings to discuss the interview guide and ensure consistency. Participants were asked to reflect on the barriers and facilitators to implementing BE changes, as well as their role(s) in supporting these changes within their organizations. The duration of the interviews varied (20–85 min) and the interviewer recorded hand-written notes. Interviews were audio recorded and transcribed verbatim by the interviewer. Transcripts from Montréal were translated to English by members of the research team and checked by the Montréal research team leader to ensure accuracy.

**Table 2:** Number of KI and VFG participants by municipality

	KI Interviews	VFG Participants	KI and VFG	Total
Vancouver	6	11	4	13
Calgary	14	12	10	16
Peel	6	9	3	12
Toronto	79	13	4	18
Montréal	7	14	0	21
**Total**	**42**	**59**	**21**	**80**

Virtual focus groups (VFGs) were conducted from July to November 2020 and the number of participants varied by municipality (Table 2). We used *Upwords* [31], a virtual platform designed to facilitate online discussion between participants, to conduct the VFGs. Focus groups were intended to be held in person but the format was modified due to COVID-19 restrictions. The online platform allowed participants to respond to questions, comment on other participants’ contributions, and was active for one week (Monday-Friday). The Consolidated Framework for Implementation Research (CFIR) [32, 33] informed the VFG interview guide. The CFIR “provides a menu of constructs that have been associated with effective implementation,” [32] which is a practical guide for examining barriers and facilitators to implementation. It is important to note that since the CFIR was used to inform the design of this study, a second iteration was developed [34]. This current work was informed by the original version [33].

Members of the research team worked with one *Upwords* staff member to modify the interview guide to accommodate the VFG format, which required participants to respond to questions in text format about their role(s) within their organization, how they are involved in BE change, and barriers and facilitators they experienced when implementing these changes. The discussions were monitored by the first author with assistance from an *Upwords* facilitator. When appropriate, participants were asked to provide additional details to their responses, as well as respond to follow-up questions from the monitoring research team member. Due to the VFGs textual format data did not require transcription and an *Upwords* staff member translated the Montréal transcripts to English, which were checked by the research team leader in Montréal to ensure accuracy. Summaries of KI interview and VFG data were written by the first author and sent back to the participants to ensure validity. Participants were given the option to review their transcript.

### Coding and analysis

Thematic analysis (TA) [35, 36] was used to code and analyze the data to determine barriers and facilitators to BE change, which also illuminated themes pertaining to HE [21–23]. The first author familiarized themself with the data by reading and re-reading the transcripts and making handwritten notes. NVivo (QSR International Pty Ltd, version 12.6.1, 2019) was used to organize the data for analysis and an inductive coding scheme was used to identify patterns across the data about barriers and facilitators and, subsequently, to highlight accessibility and HE concerns related to the BE and BE change processes. Previous handwritten notes were revisited during this phase. Once coded, the first author searched for themes and sub-themes while organizing codes accordingly. This step also included exploring “the relationship between themes and to consider how themes will work together in telling an overall story about the data” [35]. Themes and sub-themes were reviewed in relation to the coded data by the first, second, and eighth authors using a recursive process to ensure quality before being defined and named. Writing and analysis were conducted simultaneously throughout the TA process.

## Results

In their discussions of barriers and facilitators to BE change participants addressed HE concerns in four significant ways: (1) exacerbated risks for VRUs who experience marginalization; (2) inequities in the BE change process; (3) strategies for inclusive BE change consultation; and (4) BE changes to address HE concerns. While the CFIR [32, 33] was used in the design of the larger study [17], the following results are organized thematically to illustrate how HE concerns influence perceptions of BE change from the perspectives of transport and injury prevention professionals in Canadian urban municipalities.

### Exacerbated risks for VRUs who experience marginalization

Several participants described accessibility and equity concerns related the current BE in their municipality. A Peel Region (public health) participant explained,

We know that vulnerable populations (e.g., school-aged children, seniors, lower SES) are disproportionally impacted by a BE that does not support health and safety. Improving the BE should have more benefits for vulnerable populations because it’s an attempt to reduce health inequities.

This signifies an acknowledgment of the HE concerns related to an inequitable BE. It also highlights a connection between HE, the BE, and how road users’ safety is dependent on the design of the BE. This was similarly articulated by a Calgary (transportation) participant who remarked on the increased risk to non-motor vehicle road users:

Safe means more than “freedom from collision-induced physical harm.” It is an unfortunate reality that people in our society are targets of physical and emotional abuse as a result of their gender, sexual orientation, race, and social class, and cars can provide protection from risks other than being hit by another car.

This passage highlights an awareness of how pre-existing vulnerabilities intersect and are exacerbated by the current design of the BE, while also identifying the safety, protection, and privilege associated with motor vehicle use.

#### Low SES and new Canadians

Many participants described the disproportionate health risks experienced by people living in low SES neighbourhoods due to an inadequate BE and lack of road safety interventions (e.g., traffic calming). A Vancouver (public health) participant explained,

In neighborhoods that have the lowest economic status, the infrastructure is poor. There is lower prevalence of sidewalks, or separation for traffic speeds. Traffic speeds are often higher, there is more industrial than commercial traffic, and the crosswalks are not as good or signalized.

Given that low neighbourhood SES is correlated with poor overall health, this passage highlights increased vulnerability and HE concerns associated with lower SES communities.

New Canadians were also identified as a group experiencing disproportionate risk due to lack of road safety interventions and low SES, which highlights heightened HE concerns for road users with intersecting vulnerabilities. A Calgary (non-profit) participant commented on the increased risks for new Canadians with low SES: “communities that have a higher proportion of new Canadians or have a higher proportion of people experiencing low incomes, we know that they tend to be underserved when it comes to active transportation infrastructure.” This passage highlights the elevated risks experienced by people with low SES who are also new Canadians when navigating the BE in their neighborhoods.

#### Older adults and child pedestrians

Older adults and children were identified by several participants as vulnerable age groups, within pedestrians generally, negatively affected by BE inequities. For example, a Calgary (community association) participant described the effects an inequitable BE has on the health and safety of older adults:

It has contributed to social isolation, and the seniors don’t want to leave their communities because they are afraid to cross that road. And we know from traffic studies that if a senior gets hit, they’re way more likely to die because they’re more fragile.

This illustrates how an inadequate BE not only increases risks for older adults, but also discourages AT.

A Toronto (schoolboard) participant emphasized the importance of focusing on the safety needs of children “because they require a greater deal of effort to help make their communities safer for them to actively travel in,” which was affirmed by a Montréal (public health) participant: “children are more at risk of collisions in some neighbourhoods and less in others” due to the design of the BE. Further, a Vancouver (public health) participant identified children and older adults as particularly vulnerable, especially if they also have low SES: “people with lower socioeconomic status are at higher risk of injury, particularly children and the elderly.” These data illustrate the heightened awareness and concern for the health and safety of older adults and children due to the current design of the BE.

#### Accessibility & (dis)ability

People with (dis)abilities have different mobility needs, which are not always accounted for in the design of the BE. A Montréal (policy/decision-maker) participant explained how some intersections do not have the proper tactile features to guide people with vision impairments: “a visually impaired person who arrives at the intersection doesn’t like it because they lose their bearings. They are unable to decide whether to enter the intersection.” This was affirmed by another Montréal (policy/decision-maker) participant: “those who cannot see want certain types of obstacles to find their way around.” However, participants also described challenges with making the BE safe and accessible for all users. For example, “people who are in wheelchairs, who have walkers or who have a stroller, want it as flat as possible” (Montréal, policy/decision-maker), which conflicts with the needs of people with vision impairments. These data illustrate how HE concerns for VRUs are discussed and the challenges that arise when accommodating different road user needs.

### Inequities in the BE change process

Several participants discussed the BE change process and how communities can meaningfully contribute to the design and location of desired changes in their neighbourhoods. However, traditional approaches to community consultation, such as the complaints process, open houses, and petitions are not accessible to all users. As a result, the needs of some community members will be unaccounted for, which perpetuates inequality and disproportionate risk to injury for groups who experience marginalization.

#### Complaints process

A Toronto (transportation) participant explained how the current process for BE change relies on community members notifying the City about desired changes: “traffic comment requests come in and there’s a traffic calming warrant process.” Furthermore, a Peel Region (public health) participant noted how residents’ complaints directs resources within the Region: “residents may be complaining and that’s a big thing for traffic calming and changing infrastructure within a neighborhood.” However, this strategy “rewards high socioeconomic status over people who have lower socioeconomic status” (Vancouver, public health) due to the time required to engage in these processes. A Calgary (transportation) participant shared similar concerns: “often it’s the more affluent, more highly educated citizens, who know how to work with government processes. They are more likely to be demanding of City services or responses and expect a higher level of service.” A Vancouver (public health) participant explained how this process is inaccessible for groups who experience marginalization:

A lot of it is complaints driven, so if there is a community where they have the capacity to complain, or the knowledge on how to complain, they can get elevated, but of course that favors economic, more socially privileged groups. And evidence shows that those are the ones with lower risk for injury.

Similar observations were shared by a university health researcher from Vancouver describing an engagement project with school children:

The biggest challenge was that we didn’t get a lot of children from lower socio-economic and racialized groups. The commitment was considerable and parents had to fill out questionnaires too, so that could have been daunting. We need to do a better job of engaging different populations.

This demonstrates how people and children experiencing low SES can encounter barriers to existing BE change processes.

#### *Open houses and petitions*

Many participants discussed the inadequacies of open houses as a mechanism for community consultation about BE changes. A Calgary (non-profit) participant explained, “this model of having open houses and expecting people to come to you is a waste of time. You’re always going to get the same people, which is not representative.” A Vancouver (non-profit) participant expressed similar concerns with consulting neighborhood associations: “they are often a collection of a few loud voices.” Some participants also raised concerns regarding community surveys and petitions as strategies for consultation: “we had this really extensive petition process where you’d have to go door-to-door. On the one hand, it’s good because it forces the neighbours to have a conversation. But on the other hand, it’s so much work” (Calgary, transportation). This strategy also requires residents to be available and knowledgeable, assumptions that exclude some community members and groups who experience marginalization. Overall, participants were critical of consultation strategies that are not accessible for all community members.

### Strategies for inclusive BE change consultation

Several participants discussed the importance of inclusive consultation processes; specifically, the reality that each community may require a different approach to consultation, as well as different BE changes to ensure safe and accessible transportation. This was expressed clearly by a Vancouver (university) participant:

It might have to look differently for different communities; it’s not a one-size-fits-all approach. In some communities you’re not going to get anybody out to a public forum, you’re not going to get meaningful feedback, so you have to actually know what would be most meaningful for that community by having multiple pathway points for access. Maybe it’s social media, maybe it’s public forums, or maybe it’s a community event. It’s got to be diverse, and it has got to be grounded in the community.

This illustrates the importance of not only changing the BE according to the particular needs of communities, but the consultation process must also be adapted to suit the needs of equity-deserving road users.

A Montréal (non-profit) participant described strategies for successful consultation, which included continuous dialogue with the local community:

Our approach puts users at the heart of the process to ensure that they have a voice in decision-making. This does not mean that all opinions and ideas are necessarily equal or considered. However, this method promotes dialogue between the parties, encourages empathy exercises, and often allows participants to better understand the position of others, especially the most vulnerable, and the decisions that are made.

Another Montréal (policy/decision-maker) participant commented on the importance of continuous consultation with communities:

We talked to all kinds of people, we held public consultations. It’s really about talking to people and regular communication. You have to remind them that we have not forgotten, we are continuing, we are taking care of you, we are present, we take your concerns into account.

This passage highlights the positive effects of inclusive consultation practices; specifically, the importance of building rapport and continuing consultation strategies that prioritize the needs of community members.

### BE changes to address HE concerns

Many participants described how HE priorities are embedded in their work. A Vancouver (public health) participant explained, “VRUs are absolutely prioritized, and within these users, further prioritization is accorded to elderly and child/youth VRUs and to marginalized or socially disadvantaged road users. Social equity is a fundamental consideration in all our work.” Further, a Montréal (university) participant stressed the importance of making BE changes that ensure the safety of all users: “these measures lead to lasting changes that protect all individuals, regardless of age, gender, health, behaviour or socioeconomic level.” In addition, a Peel Region (transportation) participant explained how they used walking speed data to increase pedestrian crossing times:

We’re looking at a review of walking speeds of pedestrians; the amount of time that’s allocated to pedestrians crossing our roadways. And there’s a sensitivity, obviously, to areas where there are schools and seniors. So, some of our signal timings have been modified to provide for additional timing in those specific areas.

Other changes described by participants included “connected pedestrian networks (e.g., ensuring sidewalks don’t end abruptly)” (Toronto, schoolboard), “wider sidewalks, room to pass, room for mobility devices” (Vancouver, non-profit), and “ensuring there is lighting along the pathway/walkway to transit stops to encourage users to feel safe and use the pathway regardless of the time of day” (Peel Region, public health).

## Discussion

The results of this study provide insight from Canadian transport and injury prevention professionals regarding the urgent need for BE changes that enhance the safety of equity-deserving groups [18] who experience marginalization. They demonstrate a need to modify BE change consultation approaches and to embed HE principles [21, 25] within BE change strategies and actions. Further, our results highlight the need for upstream policy changes that not only acknowledge VRUs’ dependency on the BE for health [6, 13, 14], but also address these concerns by reducing barriers to BE changes, such as motor vehicle prioritization [17]. Such changes would enhance safety and reduce injury rates for road users made vulnerable by the current BE (VRUs).

### Equity-focused BE changes

Participants identified inadequacies with the current BE and how some equity-deserving groups [18] experience disproportionate risk to injury [27, 28], as well as barriers to accessing green spaces and key amenities such as “places to recreate, learn, work, shop and buy healthier food” [3]. Further, neighbourhoods with low SES experience increased health risks due to the lack of BE infrastructure to support VRUs [19], risks which are exacerbated for specific vulnerable groups such as older adults, children, and people with (dis)abilities. In addition, people with (dis)abilities have specific, and sometimes conflicting, BE needs, such as those with vision impairments and those who wheel, requiring more intensive HE-focused BE change strategies. Lastly, vulnerable groups, such as new Canadians, experience disproportionate health risks and “understanding how cultural obstacles are intermingled with economic status is key to achieving greater health equity” [3]. In other words, there is a need for more inclusive mechanisms that enable the participation of groups who experience marginalization in BE change consultation in order for the BE to reflect and support their safety needs.

Identification of vulnerable groups and understanding their specific needs is pivotal to HE action through BE change. For example, when public health practitioners in Ontario (two of the five regions included in this study) develop road safety programs, they are recommended to use strategies to ensure equitable consultation processes as per the Ontario Public Health Standards, Health Equity Guideline [25]. The recommendation focuses on “priority populations,” defined as “those that are experiencing and/or at increased risk of poor health outcomes due to the burden of disease and/or factors for disease; the determinants of health, including the social determinants of health; and/or the intersection between them” [25]. Our results identify changes that would enhance the safety of equity-deserving groups [18], which is a critical step in improving the health of road users who experience marginalization.

### HE and community consultation

As noted above, there is a variation in BE needs for different equity-deserving groups [18], aligning with the literature in this area [4, 27, 28]. To gain an understanding of these needs and the BE changes required, consultation strategies designed to promote access and participation are key [25, 37, 38]. Participants identified traditional *complaints processes*, *open houses*, and *petitions* as inadequate strategies for community consultation about BE changes because they are not equally accessible for equity-deserving groups [39]. For example, complaints processes require time and knowledge of one’s local political system, which are not universally accessible for people with low SES and/or new Canadians. Further, open house schedules do not accommodate the typically busy schedules of people with low SES. Applying a HE lens to this process illuminates these disparities and emphasizes the requirement of seeking “opportunities to engage priority populations in the design and implementation of assessment, surveillance, research, and evaluation processes” [25], similar to the co-design process explained in our results.

Participants emphasized the need for more inclusive consultation strategies. Inclusion can be defined as “the practice of ensuring that all individuals are valued and respected for their contributions and are equally supported” [40]. This aligns with a HE approach, which “must be grounded in an understanding of a particular community’s values, identities, lived experiences, as well as the economic, social, environments, and political context” [25]. It is important to understand such nuances because “individuals, communities, and populations may experience these factors differently based on social or economic conditions” (p. 5). Initiatives involving communities and equity-deserving groups [18] in BE change can potentially address health concerns and make HE a priority; specifically, it has the potential to reduce inequalities “not only by impacting social and environmental determinants of health but also by building participatory decision-making opportunities to empower communities” [16].

## Limitations

A limitation of our study is that participants were not asked directly about HE and accessibility as it pertains to the BE. Thus, participants may not have provided as detailed responses regarding the influence of HE concerns in their BE work. Further, our study employed sampling techniques [29, 30] to select participants, which may have elicited more similar responses regarding barriers and facilitators to BE change compared with random recruitment. Given that semi-structured interviews were conducted by different members of the research team across the five municipalities, follow-up and probing questions differed slightly and resulted in varying participant responses. Further, interviewers met to discuss the interview guide prior to conducting interviews but no additional steps were taken to ensure consistency. VFGs allowed participants to respond to interview questions and each other via text [31], which did not allow for in-person conversation and may have influenced responses. Finally, the positionality of each interviewer may have influenced participants’ contributions.

## Conclusion

The results of this study illustrate how HE concerns influence perspectives of the BE and BE change processes from the perspectives of urban Canadian transport and injury prevention professionals. Highlighting these participants’ perspectives of HE in relation to the BE and BE change makes an important contribution to the fields of transport, injury prevention, and public health research that policy-makers in the Canadian context ought to consider when making decisions about BE change. Further, the design and results of this study support the need for additional research focusing on the perspectives of injury prevention and transport professionals with regards to BE changes and decision-making in municipalities within Canada and abroad. Drawing on the experiences of professionals working in, and across, these sectors also shows how HE concerns and BE change are not contained within a single sector. Alternatively, efforts to improve BE conditions and the health and safety of road users exist across sectors, which bolsters the need for cross-sectoral collaboration and collective efforts to ensure that HE concerns are addressed on multiple fronts.

These results also make a compelling case for upstream HE-oriented policy changes [25] that influence geometric modifications, promote local community consultation strategies [26, 37], and require the collective efforts of professionals across sectors [25, 41, 42], which are necessary in order to enhance the safety of equity-deserving [18] groups and VRUs. Thus, future policy-makers ought to include the perceptions and views of injury prevention and transport professionals when making changes to existing BE and health policies, as well as in their efforts to engage equity-deserving [18] communities in their consultation processes. Lastly, this research contributes to ongoing efforts to improve the health of all Canadian road users; specifically, changing the BE and accommodating varying needs in BE change strategies is an actionable way to implement HE principles and support the health and safety of equity-deserving road users.

## Data Availability

The datasets generated and/or analysed during the current study are not publicly available due to the fact that they are individual interview transcripts that are the property of the participant but are available from the corresponding author on reasonable request.
